# Integrated Bulk and Single‐Cell RNA‐Seq Analysis Reveals Transcriptional Activation of PTGS2 by FOS in Progression From T2DM to T2DM‐Associated NAFLD

**DOI:** 10.1111/jcmm.71182

**Published:** 2026-05-10

**Authors:** Rong Lin, Leqin Xu, Yi Zhou, Yanjing Fan, Huan Xie, Wanzhang Li, Tianchi Hu, Chao Liu

**Affiliations:** ^1^ Department of Endocrinology Affiliated Hospital of Integrated Traditional Chinese and Western Medicine for Nanjing University of Chinese Medicine Nanjing China; ^2^ Department of Endocrinology Xiamen Hospital of Traditional Chinese Medicine (Xiamen Hospital, Beijing University of Chinese Medicine) Xiamen China; ^3^ Xiamen Hospital of Traditional Chinese Medicine (Xiamen Hospital, Beijing University of Chinese Medicine) Xiamen China

**Keywords:** apoptosis, biomarkers, FOS, inflammation, nonalcoholic fatty liver disease, PTGS2, type 2 diabetes mellitus

## Abstract

Type 2 diabetes mellitus (T2DM) and nonalcoholic fatty liver disease (NAFLD) frequently coexist, exacerbating disease burden. However, the molecular mechanisms underlying the progression from T2DM to T2DM‐associated NAFLD remain unclear. This study investigated the regulatory function of FOS‐mediated PTGS2 activation in this transition. We integrated bulk RNA‐seq data from GEO, single‐cell transcriptomic data and transcriptomes from patients with T2DM‐associated NAFLD. Differentially expressed genes were identified using the limma package, and T2DM‐related gene modules were defined by weighted gene co‐expression network analysis. LASSO regression and random forest identified 14 candidate genes, with PTGS2 and FOS prioritised. Single‐cell analysis showed increased FOS and PTGS2 expression in monocytes, CD8^+^ T cells and Kupffer cells. Transcription factor prediction and dual‐luciferase assays confirmed that FOS directly binds the PTGS2 promoter and drives its transcription. In vitro, FOS silencing decreased PTGS2 expression, cytokine secretion and apoptosis under high‐glucose and free fatty acid conditions, whereas PTGS2 overexpression exacerbated inflammation and apoptosis independently of FOS expression. These findings demonstrate that FOS transcriptionally activates PTGS2, contributing to hepatic inflammation and apoptosis during the progression from T2DM to NAFLD. PTGS2 may serve as a promising biomarker and therapeutic target for T2DM‐associated NAFLD.

**Abbreviations**
ALT: alanine aminotransferase
AST: aspartate aminotransferase
BAX: BCL2‐associated X protein
BCL‐2: B‐cell lymphoma 2
BMI: body mass index
CAT: catalase
COX‐2: cyclooxygenase‐2
DEGs: differentially expressed genes
DEGs‐T: differentially expressed target genes
DEGs‐TF: differentially expressed transcription factors
ELISA: enzyme‐linked immunosorbent assay
FFA: free fatty acids
FOS: FBJ murine osteosarcoma viral oncogene homologue
FPG: fasting plasma glucose
GO: Gene Ontology
HbA1c: glycated haemoglobin
KEGG: Kyoto Encyclopedia of Genes and Genomes
LASSO: least absolute shrinkage and selection operator
MDA: malondialdehyde
NAFLD: non‐alcoholic fatty liver disease
NF‐κB: nuclear factor kappa‐light‐chain‐enhancer of activated B cells
PTGS2: prostaglandin‐endoperoxide synthase 2
RNA‐seq: RNA sequencing
ROS: reactive oxygen species
scRNA‐seq: single‐cell RNA sequencing
SOD: superoxide dismutase
T2DM: type 2 diabetes mellitus
UMAP: uniform manifold approximation and projection
WGCNA: weighted gene co‐expression network analysis

## 1 Introduction

Type 2 diabetes mellitus (T2DM) is one of the most prevalent metabolic disorders worldwide. Persistent hyperglycemia can lead to multisystem damage, affecting the cardiovascular system, kidneys and liver [1]. Epidemiological studies have shown that the prevalence of NAFLD in patients with T2DM reaches up to 65% [2]. T2DM and NAFLD share a complex bidirectional relationship: T2DM promotes the onset and progression of NAFLD through mechanisms such as insulin resistance, chronic low‐grade inflammation and lipotoxicity [3]; conversely, the presence of NAFLD worsens glycemic control and increases the risk of adverse clinical outcomes in patients with T2DM [4]. However, early detection of T2DM combined with NAFLD remains challenging due to the invasive nature, limited sensitivity and poor accuracy of current diagnostic tools [5]. In addition, effective treatment strategies are lacking and specific therapeutic agents remain unavailable, which severely compromises patient outcomes [6]. Therefore, identifying key biomarkers involved in the progression from T2DM to T2DM combined with NAFLD and elucidating their underlying molecular mechanisms is essential for improving early diagnosis and targeted intervention.

Prostaglandin‐endoperoxide synthase 2 (PTGS2, also known as COX‐2) is an inducible cyclooxygenase involved in inflammation, lipid metabolic dysregulation and apoptosis [7]. Previous studies have shown that PTGS2 is upregulated in both T2DM and NAFLD, where it contributes to disease progression by promoting pro‐inflammatory cytokine release and tissue injury [8, 9]. However, its expression pattern and biological role in the progression from T2DM to T2DM combined with NAFLD remain poorly understood. PTGS2 transcription is regulated by several upstream transcription factors, including AP‐1, RUNX1 and NF‐κB [10, 11, 12].

Among these, FBJ murine osteosarcoma viral oncogene homologue (FOS), a key component of the AP‐1 complex, has been implicated in metabolic and inflammatory disorders. Previous studies have shown that FOS is associated with insulin resistance, lipid metabolism disorders and chronic inflammation [13, 14, 15]. In addition, c‐Fos has been reported to promote hepatic lipid accumulation and aggravate liver injury in NAFLD [16], suggesting that it may participate in the pathological progression of metabolic liver disease. Given that FOS is a potential upstream regulator of PTGS2 and that both genes are closely linked to inflammatory and metabolic dysfunction, we hypothesised that FOS may transcriptionally regulate PTGS2 during the progression from T2DM to T2DM combined with NAFLD. However, this regulatory relationship and its functional significance under the comorbid condition have not yet been systematically investigated.

Given the crucial roles of FOS and PTGS2 in T2DM and NAFLD, this study aims to investigate whether FOS regulates PTGS2 transcriptionally and thereby contributes to the progression from T2DM to T2DM combined with NAFLD. By integrating bulk RNA‐seq and single‐cell transcriptomic data and combining differential expression analysis, machine learning, transcription factor prediction and mechanistic validation, we characterise the role of the FOS–PTGS2 axis in immune and inflammatory regulation. Further in vitro experiments are conducted to confirm its function in hepatocellular inflammation and apoptosis, thereby revealing its potential mechanistic contribution and biological relevance in the progression of T2DM to NAFLD. These findings suggest that PTGS2 may serve as a promising therapeutic target and biomarker for T2DM combined with NAFLD.

## 2 Materials and Methods

### 2.1 Clinical Sample Collection and Processing

A total of 22 participants were recruited from the Department of Endocrinology, Xiamen Hospital of Traditional Chinese Medicine, including 12 patients with T2DM and 10 patients with T2DM combined with NAFLD. The diagnosis of T2DM was based on either glycated haemoglobin (HbA1c) ≥ 6.5%, fasting plasma glucose (FPG) ≥ 7.0 mmol/L or both. NAFLD was diagnosed by imaging evidence of hepatic steatosis (e.g., ultrasound) in the absence of significant alcohol consumption. Exclusion criteria included: patients with type 1 diabetes; individuals with comorbidities such as hypertension, cardiovascular or cerebrovascular disease, autoimmune disease, chronic kidney disease or psychiatric illness; individuals with a history of severe allergies or drug hypersensitivity; pregnant or lactating women; individuals who had participated in other clinical trials within the previous 3 months; excessive alcohol consumers (≥ 140 g/week for men or ≥ 70 g/week for women); and patients with other forms of chronic liver disease. The baseline clinical characteristics of the enrolled participants are summarised in Table 1.

**TABLE 1:** Clinical characteristics of participants with T2DM and T2DM combined with NAFLD.

	T2DM (*n* = 10)	T2DM combined with NAFLD (*n* = 12)	*p*
Age (years)	46.7 ± 6.1	50.3 ± 6.1	0.26
Sex (male/female)	6/4	6/6	—
FPG (mmol/L)	8.1 ± 0.4	9.2 ± 0.7	< 0.05
HbA1c (%)	7.1 ± 0.4	8.1 ± 0.3	< 0.001
BMI	22.7 ± 1.1	28.3 ± 1.6	< 0.01
AST (U/L)	20.8 ± 5.1	40.2 ± 8.5	< 0.001
ALT (U/L)	22.6 ± 6.3	39.7 ± 9.3	< 0.01

Peripheral blood samples were collected from all 22 participants. Among them, blood samples from 6 T2DM patients and 5 patients with T2DM combined with NAFLD were subjected to transcriptome sequencing. RNA extraction and high‐throughput sequencing were performed by Shanghai HuiXu Biotechnology Co. Ltd., and the resulting data were used for subsequent bioinformatics analysis. The remaining samples were used for quantitative PCR (qPCR) to validate the expression levels of key candidate genes.

### 2.2 Data Collection and Data Processing

The microarray dataset GSE189005 related to T2DM was obtained from the Gene Expression Omnibus (GEO) database and used for differential expression analysis, weighted gene co‐expression network analysis (WGCNA) and functional enrichment analysis. In addition, bulk RNA sequencing data generated from peripheral blood samples of patients with T2DM and T2DM combined with NAFLD enrolled in this study were included for integrative analysis. To validate the findings at the single‐cell level, publicly available human single‐cell RNA sequencing (scRNA‐seq) datasets associated with T2DM (GSE255566, GSE268210, GSE280401) and NAFLD (GSE136103, GSE190487, GSE235079) were incorporated. All datasets were processed in the R environment, including normalisation, batch effect correction and gene annotation conversion, to ensure comparability and reliability across analyses. Detailed information on the datasets is provided in Table 2.

**TABLE 2:** Dataset information applied for bulk and single‐cell transcriptome analyses in the study.

Dataset	Disease	Samples	Source types	Experiment type
GSE189005	T2DM	9 T2DM patients; 36 normal controls	Peripheral blood	Microarray
RNA‐seq data	T2DM; T2DM+NAFLD	6 T2DM patients; 5 T2DM+NAFLD patients	Peripheral blood	Microarray
GSE255566	T2DM	3 T2DM patients; 3 normal controls	Peripheral blood	Single‐cell
GSE268210	T2DM	10 T2DM patients	Peripheral blood	Single‐cell
GSE280401	T2DM	2 T2DM patients; 2 normal controls	Peripheral blood	Single‐cell
GSE136103	NAFLD	2 NAFLD patients; 5 normal controls	Liver tissue	Single‐cell
GSE190487	NAFLD	1 NAFLD patients	Liver tissue	Single‐cell
GSE235079	NAFLD	3 NAFLD patients	Liver tissue	Single‐cell

### 2.3 Differential Expression and Functional Enrichment Analysis

Differential expression analysis was performed using the ‘limma’ package [17] to compare T2DM patients with healthy controls and patients with T2DM combined with NAFLD against those with T2DM alone. For the bulk RNA‐seq data, raw gene expression values were normalised as transcripts per million (TPM) to account for differences in sequencing depth and gene length across samples and the resulting TPM matrix was used for downstream differential expression analysis. Genes with an absolute |log2FC| > 1 and an adjusted *p*‐value < 0.05 were considered significantly differentially expressed. Volcano plots and heatmaps were generated for visualisation. Subsequently, Gene Ontology (GO) and Kyoto Encyclopedia of Genes and Genomes (KEGG) enrichment analyses were conducted using the ‘clusterProfiler’ package to assess the biological processes (BP), cellular component (CC), molecular functions and signalling pathways associated with the differentially expressed genes [18]. Pathways with *p*.adjust < 0.05 were deemed significantly enriched, and results were visualised using the ‘ggplot2’ package.

### 2.4 Weighted Gene Co‐Expression Network Analysis

In order to identify gene co‐expression modules associated with T2DM, a weighted gene co‐expression network was constructed using the ‘WGCNA’ package [19] based on the GSE189005 dataset. A similarity matrix was first generated from the gene expression data and transformed into an adjacency matrix using an appropriate soft‐thresholding power to approximate scale‐free topology. Hierarchical clustering combined with dynamic tree cutting was then used to detect gene modules. The correlation between each module and the T2DM phenotype was calculated, and modules with strong associations were further analysed. Key modules were identified based on the relationship between module membership (MM) and gene significance (GS), providing a basis for the selection of functionally relevant genes.

### 2.5 Feature Gene Selection Using Machine Learning

To further narrow down candidate genes, the intersection of DEGs from T2DM, genes from T2DM‐related WGCNA modules and DEGs from T2DM combined with NAFLD was computed to obtain a core gene set, which was visualised using a Venn diagram. Based on this set, two commonly used machine learning algorithms were applied for feature selection. LASSO regression was performed using the ‘glmnet’ package [20] with the optimal regularisation parameter (*λ*) determined by 10‐fold cross‐validation. A random forest model was constructed using the ‘randomForest’ package, and feature importance was assessed with reference to model performance under a 10‐fold cross‐validation strategy [21]. The intersection of genes selected by both methods was considered robust and visualised with a Venn diagram.

### 2.6 Single‐Cell RNA‐Seq Data Processing and Analysis

This study integrated three human scRNA‐seq datasets related to T2DM and three related to NAFLD. Standardised preprocessing was conducted using the Seurat (v5) workflow [22]. For each dataset, quality control was performed independently. Cells were retained if they met the following criteria: mitochondrial gene content (percent.mt) < 10%, haemoglobin gene content (percent.HB) < 3%, number of detected genes (nFeature_RNA) between 300 and 5000, and UMI counts (nCount_RNA) > 1000, with the top 3% of cells with abnormally high UMI counts excluded. SCTransform was applied to normalise and stabilise variance across samples, and highly variable genes were identified for dataset integration. Harmony was subsequently used to correct for batch effects across samples in the principal component (PC) space. Dimensionality reduction was performed using Uniform Manifold Approximation and Projection (UMAP), and clustering was conducted based on the *k*‐nearest neighbour (KNN) graph. Following clustering, cell types were annotated using canonical markers referenced from the CellMarker database (http://117.50.127.228/CellMarker/) and cross‐referenced with published human single‐cell transcriptomic studies of peripheral blood and liver to ensure annotation accuracy [23, 24, 25]. Expression profiles of the key genes PTGS2 and FOS were extracted across annotated cell types. Differential expression analyses were then conducted between groups, and expression patterns were visualised using UMAP plots, dot plots and violin plots to evaluate cell type–specific expression and potential functional relevance in the context of T2DM and NAFLD.

### 2.7 Prediction and Screening of Transcription Factors and Target Genes for Key Feature Genes

To explore the transcriptional regulatory networks associated with the key feature genes PTGS2 and FOS, five widely used and representative transcription factor (TF) databases were utilised, including hTFtarget (http://bioinfo.life.hust.edu.cn/hTFtarget/), ENCODE (https://www.encodeproject.org/), GTRD (http://gtrd20‐06.biouml.org/), ChIP‐Atlas (https://chip‐atlas.org/) and JASPAR (https://jaspar.elixir.no/). These databases integrate transcriptional regulatory relationships derived from both experimental evidence and computational predictions.

Each database was queried with PTGS2 and FOS to obtain lists of putative upstream transcription factors and downstream target genes, respectively. The predicted results were then intersected with differentially expressed transcription factors (DEGs‐TF) and differentially expressed target genes (DEGs‐T) identified from the transcriptomic analysis of patients with T2DM combined with NAFLD. The intersection analyses were visualised using the ‘ComplexUpset’ package, illustrating the overlap between predicted TFs of PTGS2 and DEGs‐TF, as well as between predicted targets of FOS and DEGs‐T.

### 2.8 Cell Culture

The human normal hepatocyte cell line L02 was obtained from Fenghui Biotechnology (China). Cells were cultured in RPMI‐1640 medium supplemented with 10% fetal bovine serum (FBS) and 1% penicillin–streptomycin and maintained at 37°C in a humidified incubator with 5% CO_2_.

### 2.9 RNA Extraction and qPCR Analysis

Peripheral blood samples from patients with T2DM and T2DM combined with NAFLD were subjected to red blood cell lysis, followed by centrifugation at 3000 rpm for 10 min to collect the cell pellets. Cultured L02 cells were harvested directly by centrifugation. Total RNA was extracted from both sample types using TRIzol reagent (Sigma‐Aldrich, China), followed by chloroform extraction, isopropanol precipitation and 75% ethanol washing. The RNA pellets were then dissolved in DEPC‐treated water (Invitrogen, China), and the RNA concentration and purity were assessed spectrophotometrically. To synthesise cDNA, total RNA was subjected to reverse transcription. Quantitative real‐time PCR (qPCR) was performed using SYBR Green qPCR Master Mix (Thermo Fisher Scientific, China) on a real‐time PCR system. The relative gene expression was then calculated using the 2^2212ΔΔCt^ method, with GAPDH serving as the internal control. The primer sequences utilised in this study are enumerated in Table 3.

**TABLE 3:** Primer sequences for qPCR analysis of human genes.

Gene	Primer type	Sequence (5′→3′)
*PTGS2*	Forward primer	ATGCTCGCCCGCGCCTGCTGCTGT
	Reverse primer	ACAGCAGCAGGGCGCGGGCGAGCAT
*FOS*	Forward primer	GGGGCAAGGTGGAACAGTTAT
	Reverse primer	CCGCTTGGAGTGTATCAGTCA
*IL‐6*	Forward primer	ACTCACCTCTTCAGAACGAATTG
	Reverse primer	CCATCTTTGGAAGGTTCAGGTTG
*TNF‐α*	Forward primer	CCTCTCTCTAATCAGCCCTCTG
	Reverse primer	GAGGACCTGGGAGTAGATGAG
*MCP‐1*	Forward primer	CAGCCAGATGCAATCAATGCC
	Reverse primer	TGGAATCCTGAACCCACTTCT
*GAPDH*	Forward primer	CTGGGCTACACTGAGCACC
	Reverse primer	AAGTGGTCGTTGAGGGCAATG

### 2.10 Dual‐Luciferase Reporter Assay to Validate FOS Regulation of the PTGS2 Promoter

Based on the predicted FOS binding sites from the JASPAR database, specific primers were designed to amplify the promoter region of PTGS2 and the amplified fragments were cloned into the pGL3‐basic luciferase reporter vector to construct both wild‐type (WT) and mutant‐type (MT) reporter plasmids. All constructs were synthesised by Sangon Biotech (Shanghai, China). L02 cells were transfected with PTGS2‐WT or PTGS2‐MT plasmids using Lipofectamine 3000 (Thermo Fisher Scientific, China), and the pGL3‐basic empty vector was used as the negative control to define basal luciferase activity. To further investigate the regulatory role of FOS, a FOS‐silencing group (siFOS) and a negative control siRNA group (siNC) were co‐transfected with either PTGS2‐WT or PTGS2‐MT plasmids into L02 cells. After 24 h of transfection, cells were harvested and luciferase activity was measured using a dual‐luciferase reporter assay system (Beyotime, China) to evaluate the effect of FOS on PTGS2 promoter activity.

### 2.11 Construction of T2DM and T2DM Combined With NAFLD Cell Models

Building on previously described protocols established in HepG2 cells [26, 27, 28], this study developed modified in vitro models of T2DM and T2DM combined with NAFLD using L02 cells. To construct the T2DM model (L02‐T), L02 cells were cultured in high‐glucose medium containing 30 mmol/L D‐glucose for 24 h. Subsequently, to establish the T2DM combined with NAFLD model (L02‐TN), cells were further exposed to medium supplemented with 1 mM free fatty acids (FFA) for an additional 24 h under the same high‐glucose conditions [29, 30]. The FFA working solution was prepared by mixing oleic acid and palmitic acid at a 2:1 ratio [31]. The success of model establishment was validated by Oil Red O staining, quantitative PCR analysis of inflammatory markers (IL‐6, TNF‐α and CCL2) and apoptosis‐related genes (BAX and BCL‐2), as well as the assessment of oxidative stress indicators including malondialdehyde (MDA), superoxide dismutase (SOD), 4‐hydroxynonenal (4‐HNE) and catalase (CAT).

### 2.12 Cell Intervention and Grouping

siFOS and its negative control (siNC) were purchased from Thermo Fisher Scientific (Shanghai, China) and transfected into L02 cells using Lipofectamine 3000. After 6–8 h of transfection, the medium was replaced with high‐glucose medium for continued culture. Cells were cultured for an additional 24 h to induce the T2DM model, or cultured in high‐glucose medium supplemented with 1 mM FFA to induce the T2DM combined with NAFLD model. The PTGS2 overexpression plasmid (oePTGS2) and its negative control (oeNC), constructed in the pcDNA3.1 vector by Sangon Biotech (Shanghai, China), were transfected following the same protocol.

A total of 11 experimental groups were established: control group (L02), high‐glucose group (L02‐T), high‐glucose + FFA group (L02‐TN), high‐glucose + siNC group (L02‐T‐siNC), high‐glucose + siFOS group (L02‐T‐siFOS), high‐glucose + FFA + siNC group (L02‐TN‐siNC), high‐glucose + FFA + siFOS group (L02‐TN‐siFOS), high‐glucose + oeNC group (L02‐T‐oeNC), high‐glucose + PTGS2 overexpression group (L02‐T‐oePTGS2), high‐glucose + FFA + oeNC group (L02‐TN‐oeNC) and high‐glucose + FFA + PTGS2 overexpression group (L02‐TN‐oePTGS2). Cells were harvested 24 h after treatment for qPCR analysis, and 48 h after treatment for protein assays and functional experiments.

### 2.13 Oil Red O Staining

To assess lipid accumulation in the in vitro models, Oil Red O staining was performed on L02, L02‐T and L02‐TN cells. Cells were washed with PBS, fixed with 4% paraformaldehyde for 30 min and treated with 60% isopropanol for 5 min. Freshly prepared Oil Red O working solution was then added and incubated for 10–15 min. After staining, cells were rinsed with PBS and images were acquired under a microscope. Semi‐quantitative analysis was performed using ImageJ software.

### 2.14 Oxidative Stress Assays

To evaluate oxidative stress levels, intracellular levels of MDA, SOD, 4‐HNE and CAT were measured using commercial assay kits (Beyotime, Nanjing, China) according to the manufacturer's protocols. All assays were conducted following the kit instructions and quantified using colorimetric or ELISA‐based methods.

### 2.15 Western Blot Analysis

Total protein was extracted from cells of each treatment group and quantified using the BCA method. Equal amounts of protein were separated by SDS‐PAGE and transferred to PVDF membranes. Membranes were blocked with 5% non‐fat milk for 1 h, then incubated overnight at 4°C with primary antibodies against PTGS2 (1:1000), c‐Fos (1:500), BAX (1:1000), BCL‐2 (1:1000) and GAPDH (1:5000) (Abcam, China). After washing, membranes were incubated with HRP‐conjugated secondary antibodies (1:3000, Abcam, China) for 1 h at room temperature. Bands were visualised using ECL reagents and imaged. Densitometric analysis was performed using ImageJ software.

### 2.16 Enzyme‐Linked Immunosorbent Assay (ELISA)

Cell culture supernatants from each group were collected and centrifuged to remove debris. Levels of IL‐6 (Beyotime, China), TNF‐α (Abcam, China) and MCP‐1 (Beyotime, China) were measured using human ELISA kits, following the manufacturer's instructions. Absorbance was read at 450 nm, and cytokine concentrations were calculated based on standard curves.

### 2.17 Statistical Analysis

All bioinformatics data were analysed and visualised using R software (version 4.3.2). Experimental data were analysed and visualised using GraphPad Prism 10.0 (GraphPad Software). Clinical data were presented as mean ± standard deviation (SD). Comparisons between two groups were performed using an unpaired two‐tailed Student's *t*‐test, and multiple group comparisons were conducted using one‐way analysis of variance (ANOVA). A *p*‐value < 0.05 was considered statistically significant. All experiments were independently repeated at least three times to ensure reproducibility.

## 3 Results

### 3.1 Identification and Functional Enrichment Analysis of Differentially Expressed Genes in T2DM and T2DM Combined With NAFLD Patients

**FIGURE 1:**
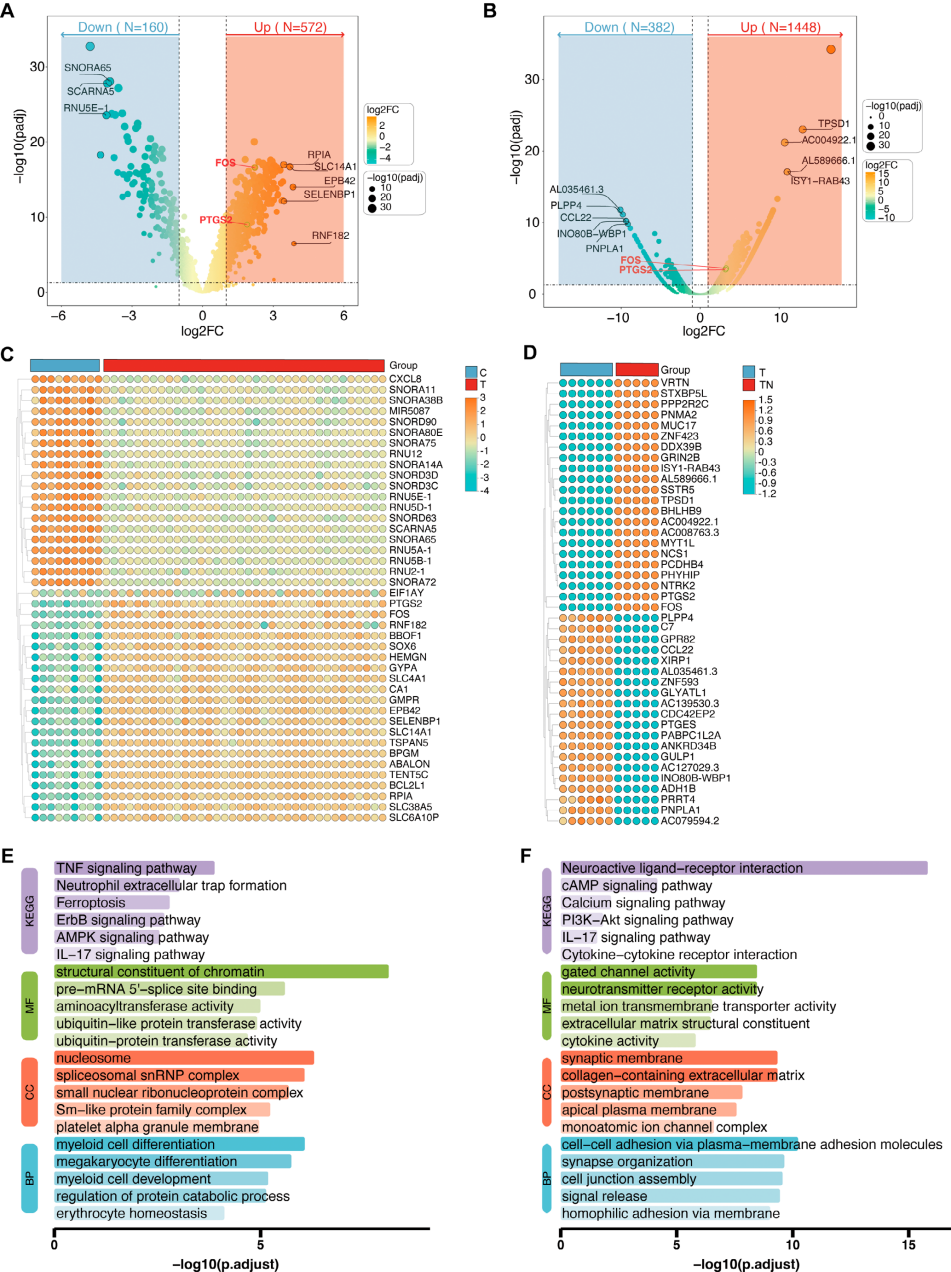
Differential expression and functional enrichment analysis in patients with T2DM and T2DM combined with NAFLD. (A) Volcano plot of differentially expressed genes (DEGs) between T2DM patients and healthy controls based on the GSE189005 dataset. (B) Volcano plot of DEGs between patients with T2DM combined with NAFLD and those with T2DM alone. (C) Heatmap showing the expression patterns of DEGs between T2DM and healthy control groups. (D) Heatmap of DEGs between the T2DM combined with NAFLD group and the T2DM group. (E) GO and KEGG enrichment analysis of DEGs in the T2DM group. (F) GO and KEGG enrichment analysis of DEGs in the T2DM combined with NAFLD group. C: Healthy control group; T: T2DM group; TN: T2DM combined with NAFLD group.

In this study, we first analysed the GSE189005 transcriptomic dataset to identify DEGs between patients with T2DM and healthy individuals. A total of 732 DEGs were identified, including 572 upregulated and 160 downregulated genes. The volcano plot and heatmap revealed significant expression differences between the T2DM and control groups (Figure 1A,C). Subsequently, transcriptomic data from peripheral blood samples of patients with T2DM and those with T2DM combined with NAFLD were analysed, resulting in the identification of 1830 DEGs, comprising 1448 upregulated and 382 downregulated genes. These results were also visualised using volcano plots and heatmaps (Figure 1B,D).

GO and KEGG enrichment analyses demonstrated that DEGs from both groups were significantly enriched in several shared inflammation‐ and signalling‐related pathways, including the TNF signalling pathway, IL‐17 signalling pathway, cytokine–cytokine receptor interaction, collagen‐containing extracellular matrix and cell adhesion molecules (Figure 1E,F). These common enrichment results suggest that chronic inflammatory activation, cytokine‐mediated immune responses and structural remodelling of cells may constitute key biological mechanisms underlying the progression from T2DM to T2DM combined with NAFLD.

### 3.2 Identification of Key Module Genes Significantly Associated With T2DM Based on WGCNA

**FIGURE 2:**
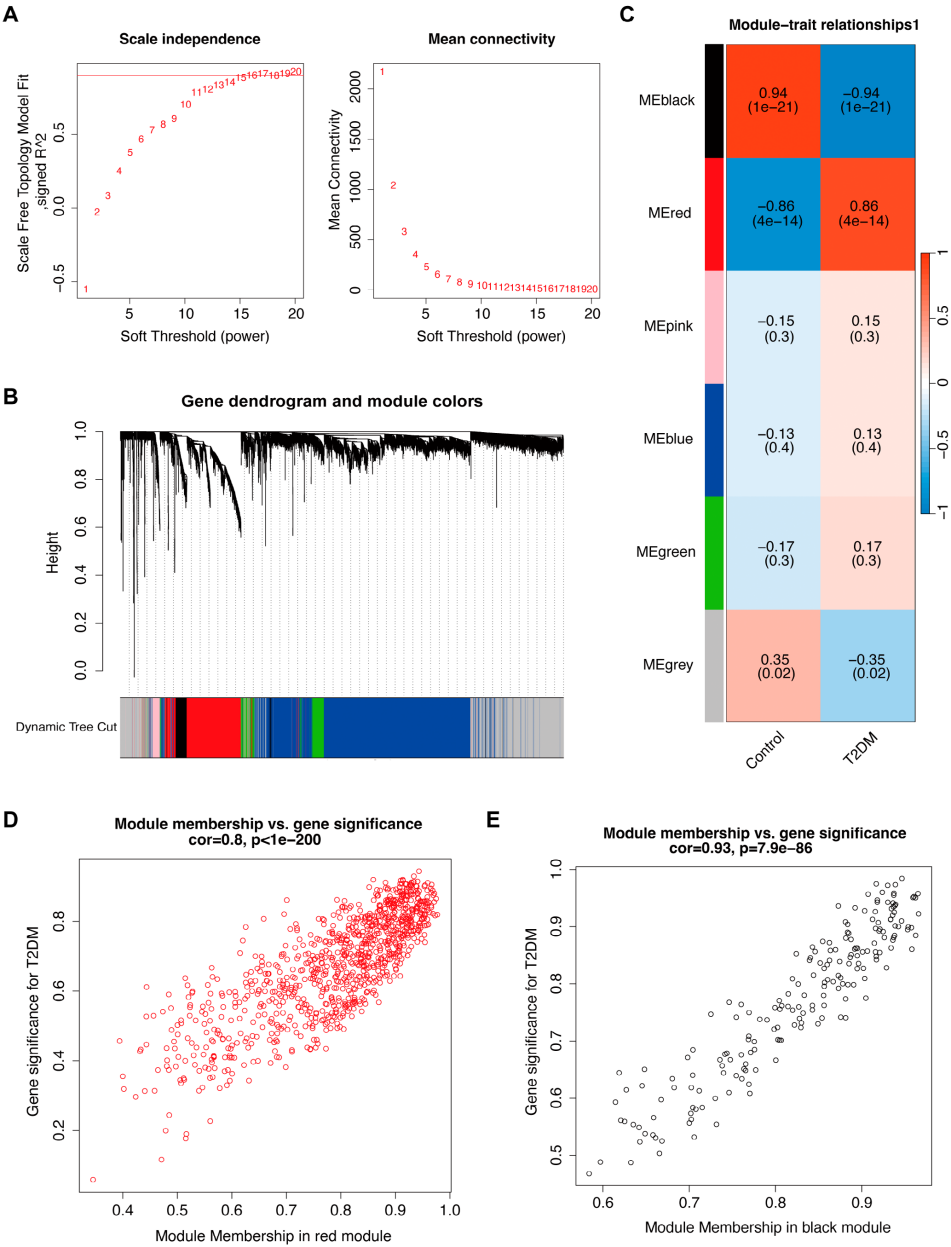
Identification of key module genes associated with T2DM using WGCNA. (A) Analysis of scale‐free topology model fit and mean connectivity across different soft‐thresholding powers. (B) Gene clustering dendrogram and module assignment based on dynamic tree cutting. (C) Heatmap of correlations between module genes and the T2DM phenotype. (D) Scatter plot showing a strong correlation between gene significance for T2DM and module membership in the red module. (E) Scatter plot showing a high correlation between gene significance for T2DM and module membership in the black module.

To identify co‐expression modules closely associated with the T2DM phenotype, WGCNA was conducted based on the GSE189005 transcriptomic dataset. As shown in Figure 2A, a soft‐thresholding power of 9 was selected to satisfy the scale‐free topology criterion. Multiple gene modules were identified following network construction (Figure 2B). Module–trait correlation analysis revealed that the black module (cor = 0.94, *p* = 1*e*−21) and the red module (cor = −0.86, *p* = 4*e*−14) exhibited strong associations with the T2DM phenotype (Figure 2C). Further analysis indicated a high positive correlation between module membership and gene significance in both the red module (cor = 0.8, *p* < 1*e*−200, Figure 2D) and the black module (cor = 0.93, *p* = 7.9*e*−86, Figure 2E). These findings suggest that these two modules may contain key regulatory genes involved in the pathogenesis of T2DM.

### 3.3 Identification of Potential Biomarkers for T2DM Combined With NAFLD

**FIGURE 3:**
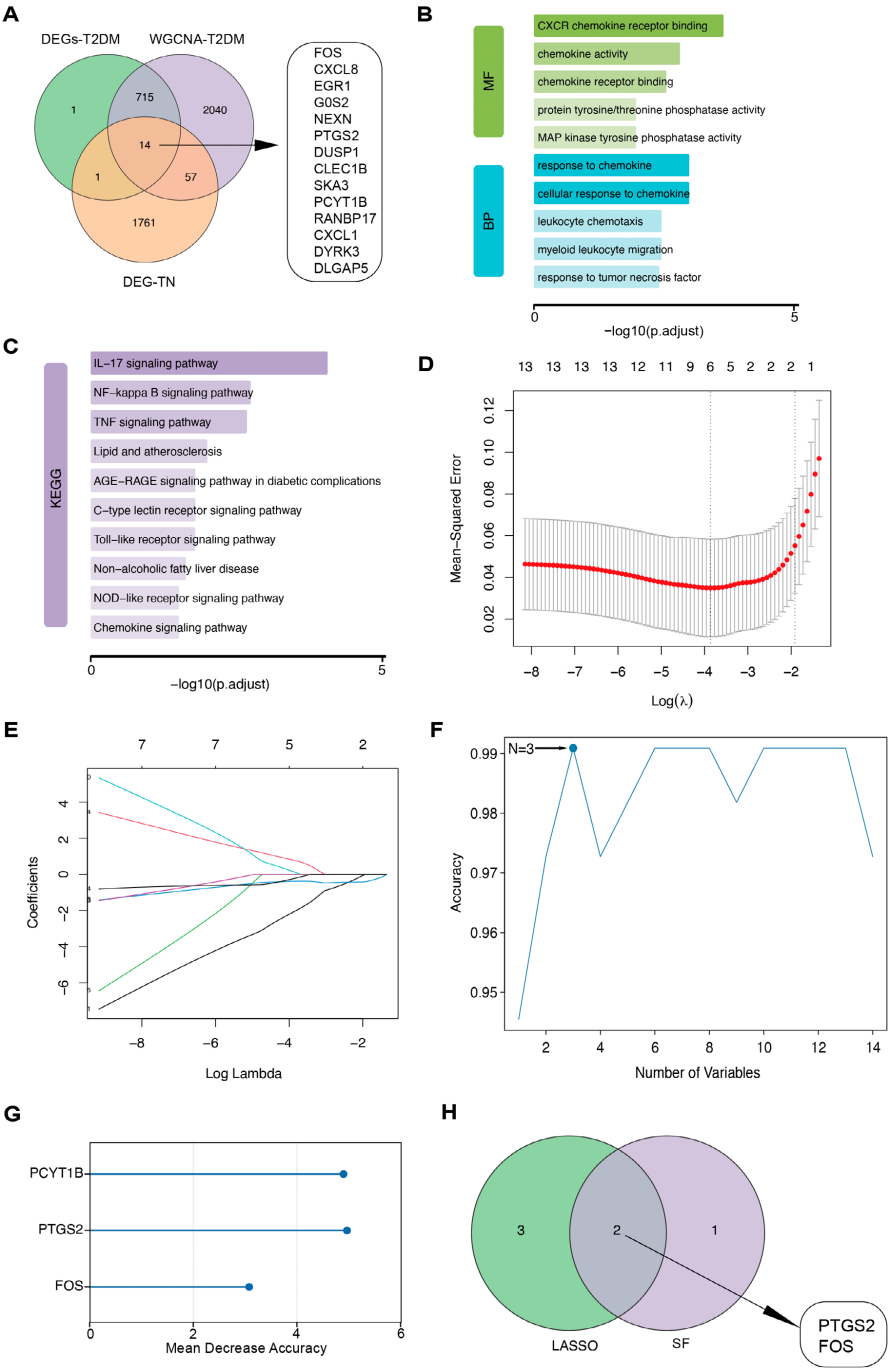
Identification of characteristic genes for T2DM combined with NAFLD via machine learning. (A) Venn diagram showing the intersection of DEGs from T2DM, genes from WGCNA modules and DEGs from T2DM+NAFLD. (B, C) GO and KEGG enrichment analyses of the intersected genes. (D) Cross‐validation curve for LASSO regression showing the relationship between lambda values and model error. (E) Coefficient profiles of genes selected by LASSO across varying lambda values. (F) Classification accuracy of the random forest model with different numbers of selected features. (G) Random Forest‐based ranking of candidate genes according to Mean Decrease Accuracy. (H) Overlap of selected genes identified by LASSO and random forest algorithms.

To identify potential regulatory genes involved in the progression from T2DM to T2DM combined with NAFLD, an intersection analysis was performed using three gene sets: DEGs from T2DM, genes from T2DM‐associated WGCNA modules and DEGs from T2DM combined with NAFLD samples. A total of 14 candidate core genes were identified (Figure 3A). Functional enrichment analysis revealed that these genes were significantly enriched in pathways related to inflammation and immune regulation, including the IL‐17, NF‐κB and TNF signalling pathways, Toll‐like receptor signalling and C‐type lectin receptor signalling, as well as metabolic disease‐related pathways such as NAFLD and atherosclerosis (Figure 3B,C), suggesting their potential involvement in the pathogenesis of T2DM combined with NAFLD.

Subsequently, two machine learning algorithms—LASSO regression and random forest—were applied to further screen for characteristic genes among the 14 candidates. LASSO regression, optimised by 10‐fold cross‐validation, identified five key genes: FOS, G0S2, PTGS2, CXCL8 and DLGAP5 (Figure 3D,E). The random forest model identified three candidate genes—PCYT1B, PTGS2 and FOS—with relatively high importance based on Mean Decrease Accuracy, as shown in Figure 3G. The intersection of both methods identified PTGS2 and FOS as robust feature genes, which may serve as potential molecular biomarkers for the progression from T2DM to NAFLD (Figure 3H).

### 3.4 FOS Promotes the Progression From T2DM to T2DM Combined With NAFLD by Transcriptionally Regulating PTGS2

**FIGURE 4:**
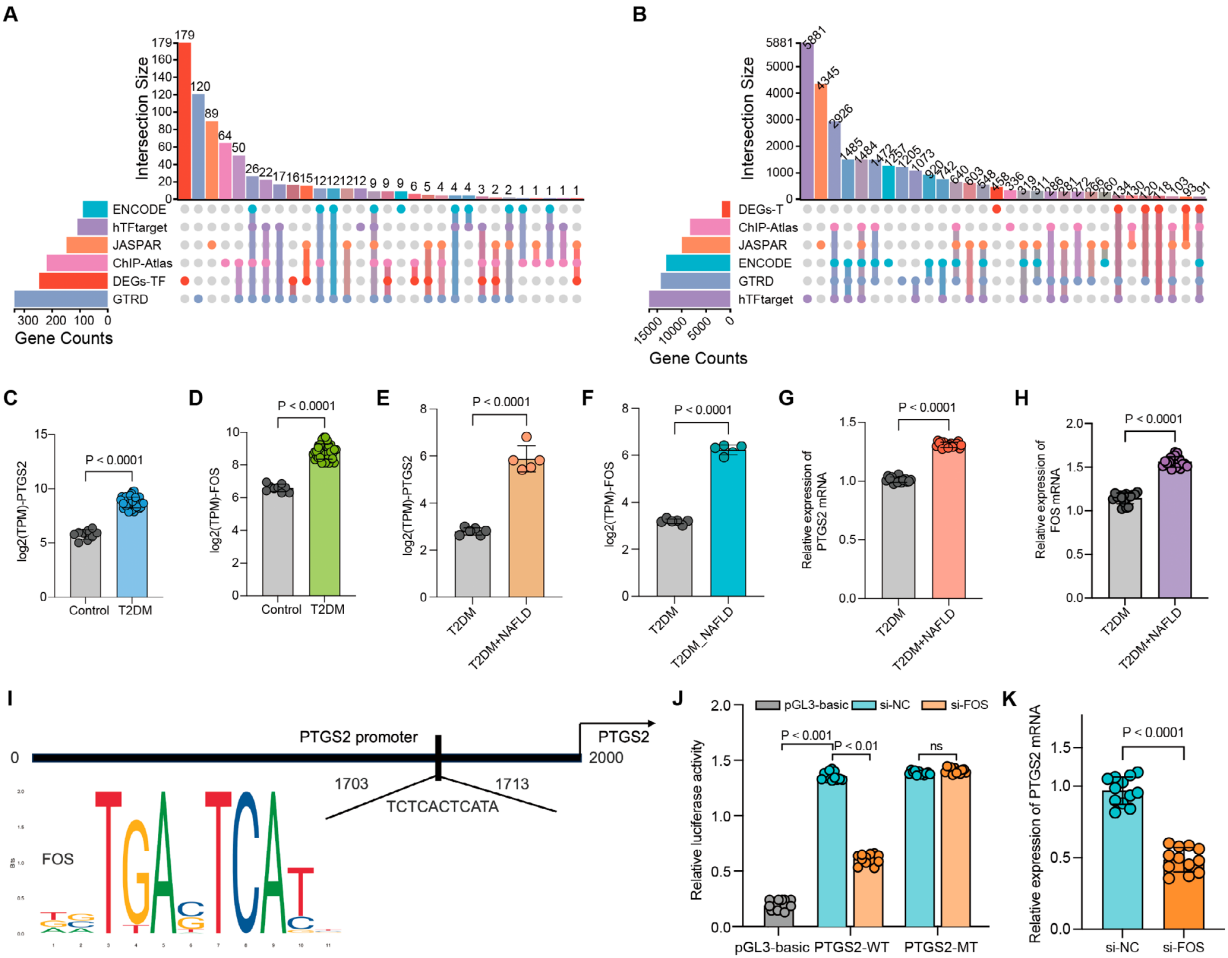
FOS regulates PTGS2 and promotes progression from T2DM to T2DM combined with NAFLD. (A) Prediction of upstream transcription factors of PTGS2 based on five databases and intersection with DEGs‐TF. (B) Intersection of FOS‐predicted target genes with DEGs‐T. (C, D) Expression levels of FOS and PTGS2 in the GSE189005 dataset. (E, F) Expression levels of FOS and PTGS2 in peripheral blood transcriptomic data. (G, H) qPCR results of FOS and PTGS2 in peripheral blood samples from clinical patients. (I) Predicted FOS binding site in the PTGS2 promoter region based on JASPAR. (J) Dual‐luciferase reporter assay of PTGS2‐WT and PTGS2‐MT promoter activity following FOS silencing, with pGL3‐basic as the baseline control. (K) Effect of FOS silencing on PTGS2 mRNA expression. Data are presented as mean ± standard deviation from three independent experiments. DEGs‐T, differentially expressed target genes; DEGs‐TF, differentially expressed transcription factors; MT, mutant type; WT, wild type.

To investigate the potential regulatory relationship between FOS and PTGS2 in T2DM combined with NAFLD, five representative transcription factor databases—hTFtarget, ENCODE, GTRD, ChIP_Atlas and JASPAR—were used to predict upstream transcription factors of PTGS2. These predicted regulators were then intersected with DEGs‐TF identified in T2DM combined with NAFLD. FOS was consistently predicted across multiple databases as a potential regulator of PTGS2 and was significantly upregulated in the DEGs‐TF set (Figure 4A). In parallel, downstream target genes of FOS were predicted and overlapped with DEGs‐T, identifying PTGS2 as a potential FOS‐regulated target (Figure 4B). Expression analysis showed that both PTGS2 and FOS were significantly upregulated in T2DM patients in the GSE189005 dataset (Figure 4C,D), as well as in T2DM combined with NAFLD samples from our independent transcriptomic data (Figure 4E,F). This upregulation was further validated in peripheral blood samples from clinical patients using qPCR (Figure 4G,H). JASPAR‐based motif prediction indicated a putative FOS binding site in the PTGS2 promoter region, suggesting the possibility of direct transcriptional regulation (Figure 4I). Dual‐luciferase reporter assays showed that, relative to the pGL3‐basic empty vector baseline, PTGS2‐WT exhibited significantly increased luciferase activity. FOS knockdown significantly reduced the activity of PTGS2‐WT, whereas no significant change was observed in PTGS2‐MT (Figure 4J). Moreover, siRNA‐mediated silencing of FOS resulted in a marked decrease in PTGS2 mRNA expression (Figure 4K). Collectively, these results indicate that FOS functions as a key transcriptional regulator of PTGS2 and may contribute to the progression from T2DM to T2DM combined with NAFLD by modulating its expression.

### 3.5 Expression Patterns of PTGS2 and FOS in Single‐Cell Transcriptomic Data of T2DM

**FIGURE 5:**
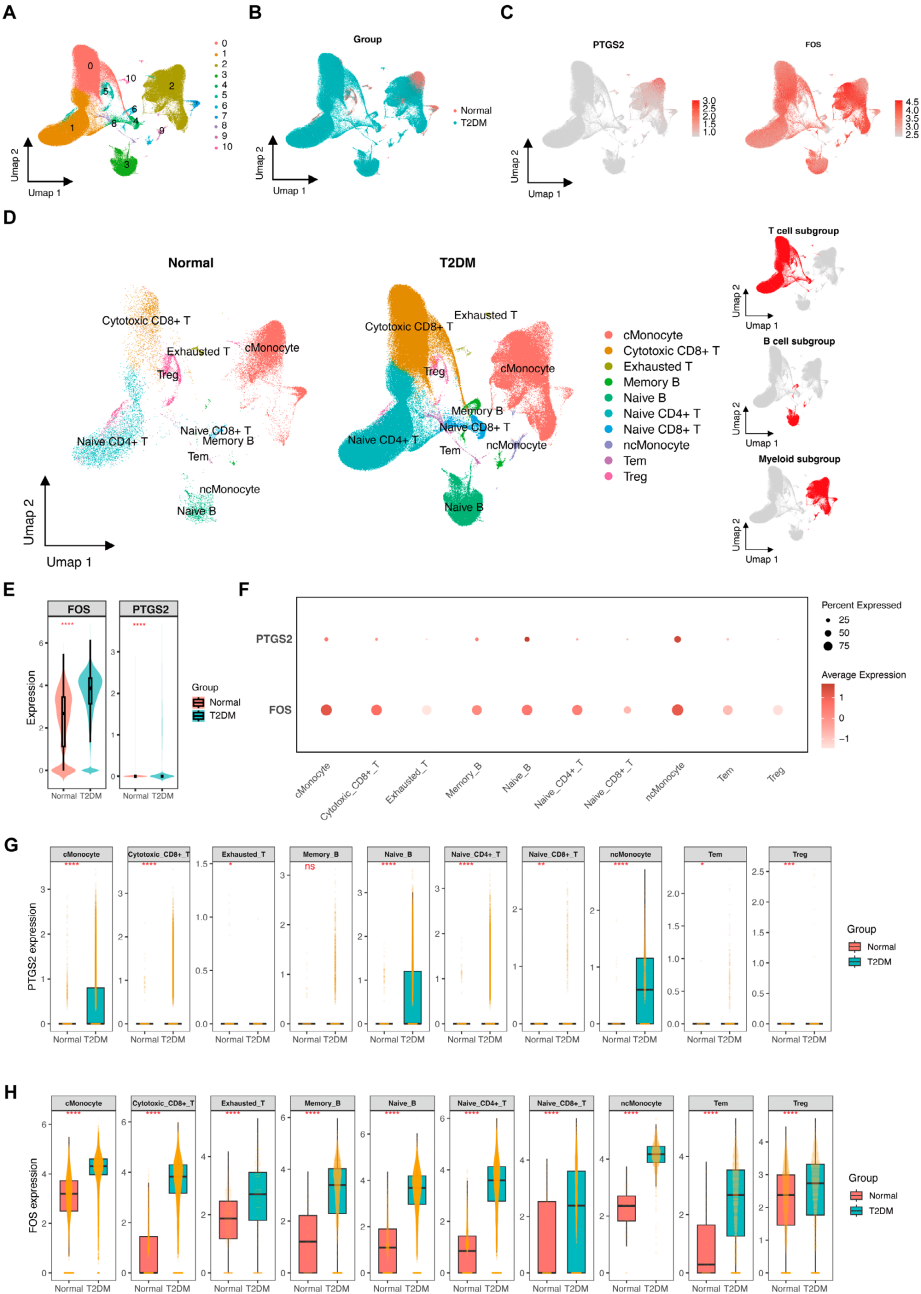
Expression patterns of PTGS2 and FOS in single‐cell transcriptomic profiles of T2DM. (A) UMAP projection of integrated single‐cell RNA‐seq data (GSE255566, GSE268210, GSE280401) showing major cell clusters. (B) Distribution of cells from Normal and T2DM groups. (C) Spatial expression of PTGS2 and FOS across the integrated cellular landscape. (D) Annotation of major immune cell subsets based on canonical markers with subgroup localisation of T cells, B cells and myeloid cells. (E) Overall expression levels of PTGS2 and FOS in Normal versus T2DM. (F) Dot plot showing average expression and percentage of expressing cells for each gene across subsets. (G, H) Comparison of PTGS2 (G) and FOS (H) expression levels between groups across immune subsets. Significance levels: ns, not significant; *p < 0.05; **p < 0.01; ***p < 0.001; ****p < 0.0001.

To investigate the cell type‐specific expression of PTGS2 and FOS in T2DM, three single‐cell RNA‐seq datasets (GSE255566, GSE268210 and GSE280401) were integrated, comprising a total of 280,683 peripheral blood cells, including 253,666 cells from T2DM patients and 27,017 from healthy controls. After quality control filtering based on gene counts, UMI counts and mitochondrial gene percentage (Figure S1A), and batch effect correction confirming effective sample integration (Figure S1B), the combined dataset was subjected to downstream analysis. After standard preprocessing and batch correction, dimensionality reduction by UMAP revealed 10 distinct cell clusters (Figure 5A) and cells were further stratified by disease status (Figure 5B). Clustering resolution was optimised based on stability across different resolutions (Figure S1C).

Cell‐type annotation based on canonical markers identified major immune cell subsets (Figure 5D), including classical monocytes (cMonocytes), memory B cells, cytotoxic CD8^+^ T cells, exhausted T cells and regulatory T cells (Tregs). Representative marker genes used for annotation are shown in Figure S1D. Violin plots demonstrated that the overall expression levels of PTGS2 and FOS were markedly elevated in T2DM compared to controls (Figure 5E). Dot plot analysis further showed that both genes were predominantly expressed in monocytes and effector T cell populations (Figure 5F). Subset‐level comparisons revealed that PTGS2 was significantly upregulated in cMonocytes, cytotoxic CD8^+^ T cells, naïve B cells and non‐classical monocytes in T2DM (Figure 5G), while FOS showed increased expression across multiple subsets, including monocytes, CD8^+^ T cells and Tregs (Figure 5H). These findings suggest that PTGS2 and FOS are selectively activated in T2DM‐associated immune cell subsets and may contribute to the immune dysregulation underlying the disease.

### 3.6 Expression Patterns of PTGS2 and FOS in Single‐Cell Transcriptomic Profiles of NAFLD Liver Tissue

**FIGURE 6:**
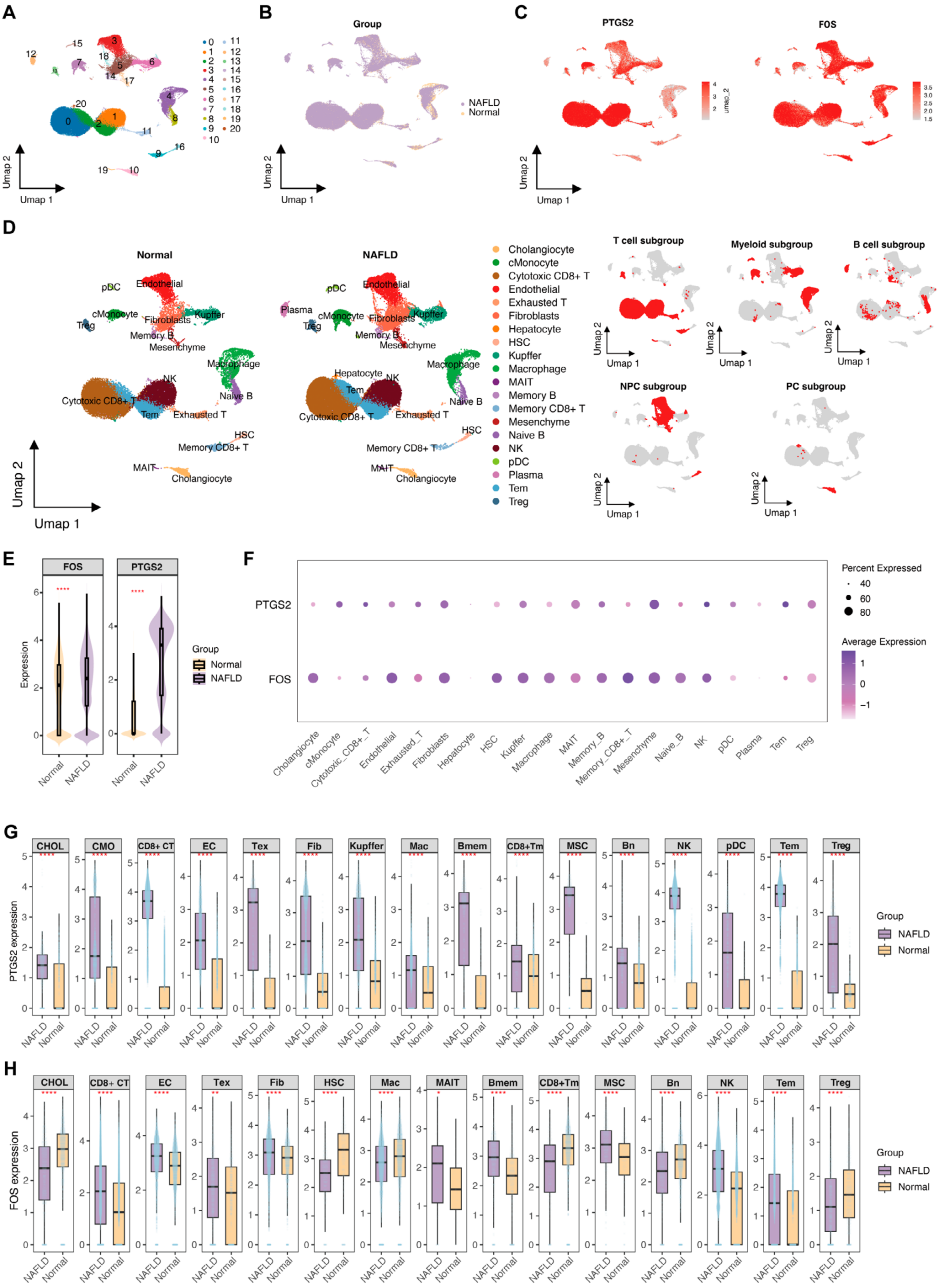
Expression patterns of PTGS2 and FOS in single‐cell transcriptomic profiles of NAFLD liver tissue. (A) UMAP projection of integrated single‐cell RNA‐seq data from human liver tissue (GSE136103, GSE190487 and GSE235079) identifying 21 major cell clusters. (B) Distribution of cells from Normal and NAFLD groups. (C) Spatial expression patterns of PTGS2 and FOS across the integrated cellular landscape. (D) Annotation of major liver‐resident immune and parenchymal cell populations based on canonical markers. (E) Violin plots showing overall expression levels of PTGS2 and FOS in Normal versus NAFLD groups. (F) Dot plot showing average expression and percentage of expressing cells for each gene across annotated cell subsets. (G, H) Comparison of PTGS2 (G) and FOS (H) expression levels between Normal and NAFLD groups across major liver cell subsets. Significance levels: ns, not significant; *p < 0.05; **p < 0.01; ****p < 0.0001. Bmem, memory B cells; CD8^+^ CT, CD8^+^ cytotoxic T cells; CHOL, cholangiocytes; CMO, classical monocytes; EC, endothelial cells; Fib, fibroblasts; KC, Kupffer cells; Mac, macrophages; MAIT, mucosal‐associated invariant T cells; pDC, plasmacytoid dendritic cells; Treg, regulatory T cells.

Building on the observed expression patterns of PTGS2 and FOS in peripheral blood immune cells of T2DM patients, this study further integrated three human liver single‐cell RNA‐seq datasets (GSE136103, GSE190487 and GSE235079), comprising a total of 76,929 cells (42,601 from NAFLD patients and 34,328 from healthy controls). After quality control filtering, including gene counts, UMI counts and mitochondrial gene percentage per cell (Figure S2A), dimensionality reduction by UMAP revealed 21 distinct clusters (Figure 6A), which clearly distinguished NAFLD samples from normal tissues based on disease status (Figure 6B). Clustering resolution optimisation was performed using clustree analysis to ensure robust and biologically meaningful clustering (Figure S2C). The effective integration of multiple samples after batch correction was confirmed by UMAP projection (Figure S2B). Spatial mapping showed increased expression of PTGS2 and FOS in NAFLD liver tissues (Figure 6C). Cell‐type annotation identified major populations (Figure 6D), including monocytes, CD8+ T cells, Tregs, Kupffer cells, cholangiocytes and endothelial cells. Representative marker genes used for annotation are shown in Figure S2D. Overall expression analysis demonstrated that both genes were significantly upregulated in the NAFLD group (Figure 6E), with elevated expression proportions across multiple cell subsets (Figure 6F).

Subset‐specific analysis revealed that PTGS2 was notably upregulated in monocytes, CD8+ T cells, Kupffer cells and endothelial cells in NAFLD samples (Figure 6G), consistent with its distribution in T2DM while also exhibiting liver‐specific enrichment. FOS showed widespread upregulation in CD8+ T cells, macrophages, Tregs, MAIT cells and B cells (Figure 6H), with a broader distribution than that observed in T2DM. Collectively, PTGS2 and FOS were highly expressed in both T2DM and NAFLD, particularly in immune‐related cell populations. Their broader expression in NAFLD liver tissue suggests a potential role in immune activation and hepatic microenvironment remodelling during the progression of T2DM and NAFLD.

### 3.7 Construction and Validation of T2DM and T2DM Combined With NAFLD Cell Models Using L02 Cells

**FIGURE 7:**
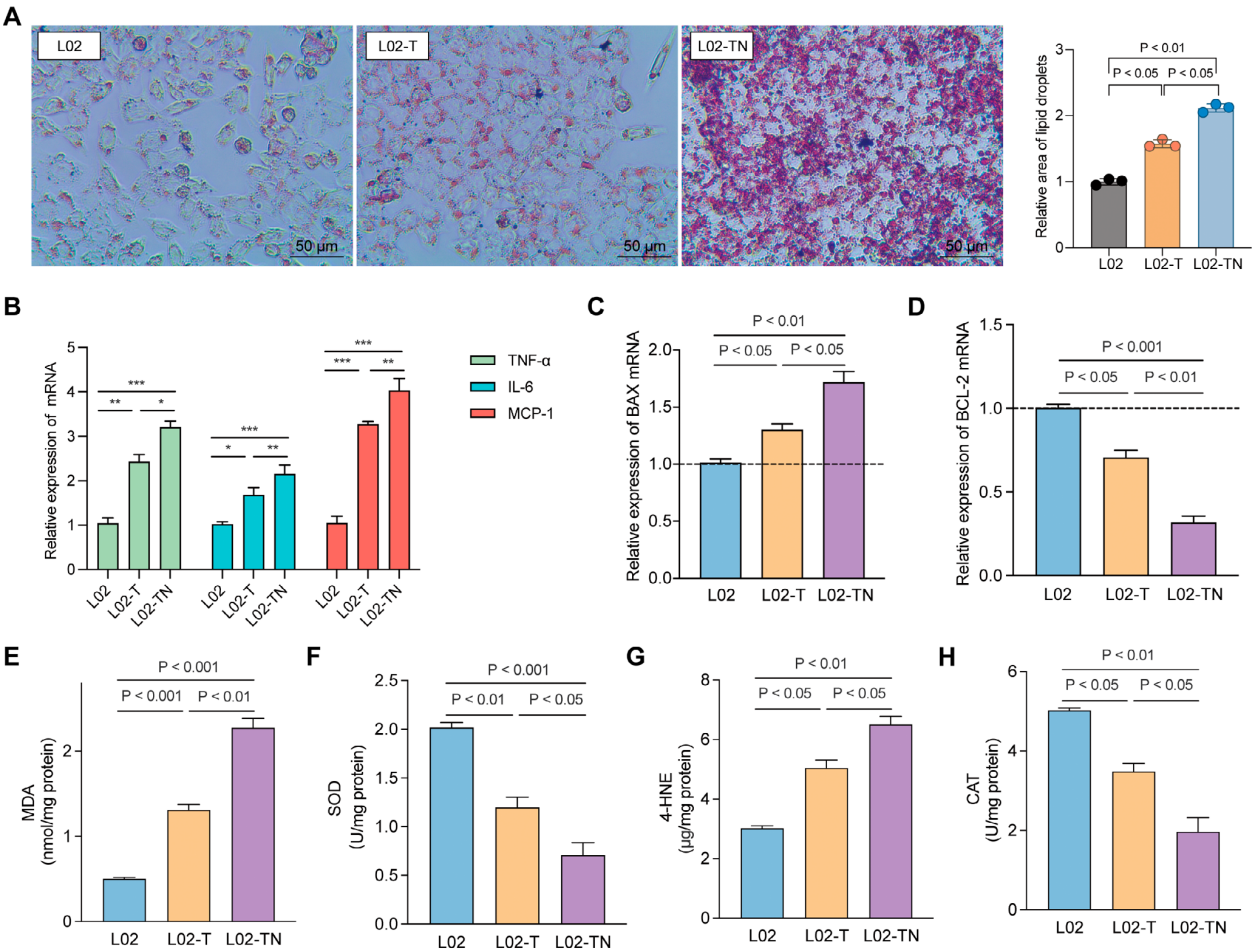
Construction and validation of T2DM and T2DM combined with NAFLD cell models in L02 cells. (A) Oil Red O staining of L02 cells under different treatment conditions and quantification of lipid droplet area. (B) mRNA expression levels of inflammatory cytokines TNF‐α, IL‐6 and MCP‐1. (C, D) mRNA expression levels of apoptosis‐related genes BAX and BCL‐2. (E–H) Levels of oxidative stress markers MDA, SOD, 4‐HNE and CAT. Data are presented as mean ± standard deviation from three independent experiments. *p < 0.05; **p < 0.01; ***p < 0.001.

To mimic the cellular microenvironment of T2DM and T2DM combined with NAFLD, L02 cells were treated with high glucose or high glucose combined with FFA. Oil Red O staining showed that, compared to the control group (L02), the high‐glucose group (L02‐T) exhibited increased lipid droplet accumulation, which was further aggravated in the high‐glucose plus FFA group (L02‐TN), indicating enhanced lipid deposition (Figure 7A). qPCR analysis revealed that the mRNA levels of inflammatory cytokines TNF‐α, IL‐6 and MCP‐1 were markedly elevated in the L02‐T group compared to L02 cells and further increased in the L02‐TN group (Figure 7B). Apoptosis‐related gene expression showed that the pro‐apoptotic gene BAX was progressively upregulated from L02‐T to L02‐TN, while the anti‐apoptotic gene BCL‐2 was downregulated, suggesting a progressive increase in apoptosis (Figure 7C,D).

Oxidative stress assessment indicated that the lipid peroxidation products MDA and 4‐HNE were significantly elevated in the model groups, with the highest levels observed in L02‐TN cells (Figure 7E,G). Conversely, the antioxidant enzymes SOD and CAT showed a decreasing trend in both L02‐T and L02‐TN groups (Figure 7F,H). These findings demonstrate that high glucose and high glucose combined with FFA successfully induced T2DM and T2DM combined with NAFLD phenotypes in L02 cells, characterised by excessive lipid accumulation, inflammation, enhanced apoptosis and oxidative stress imbalance.

### 3.8 In Vitro Validation of FOS‐Mediated Regulation of PTGS2 in the Progression From T2DM to T2DM Combined With NAFLD

**FIGURE 8:**
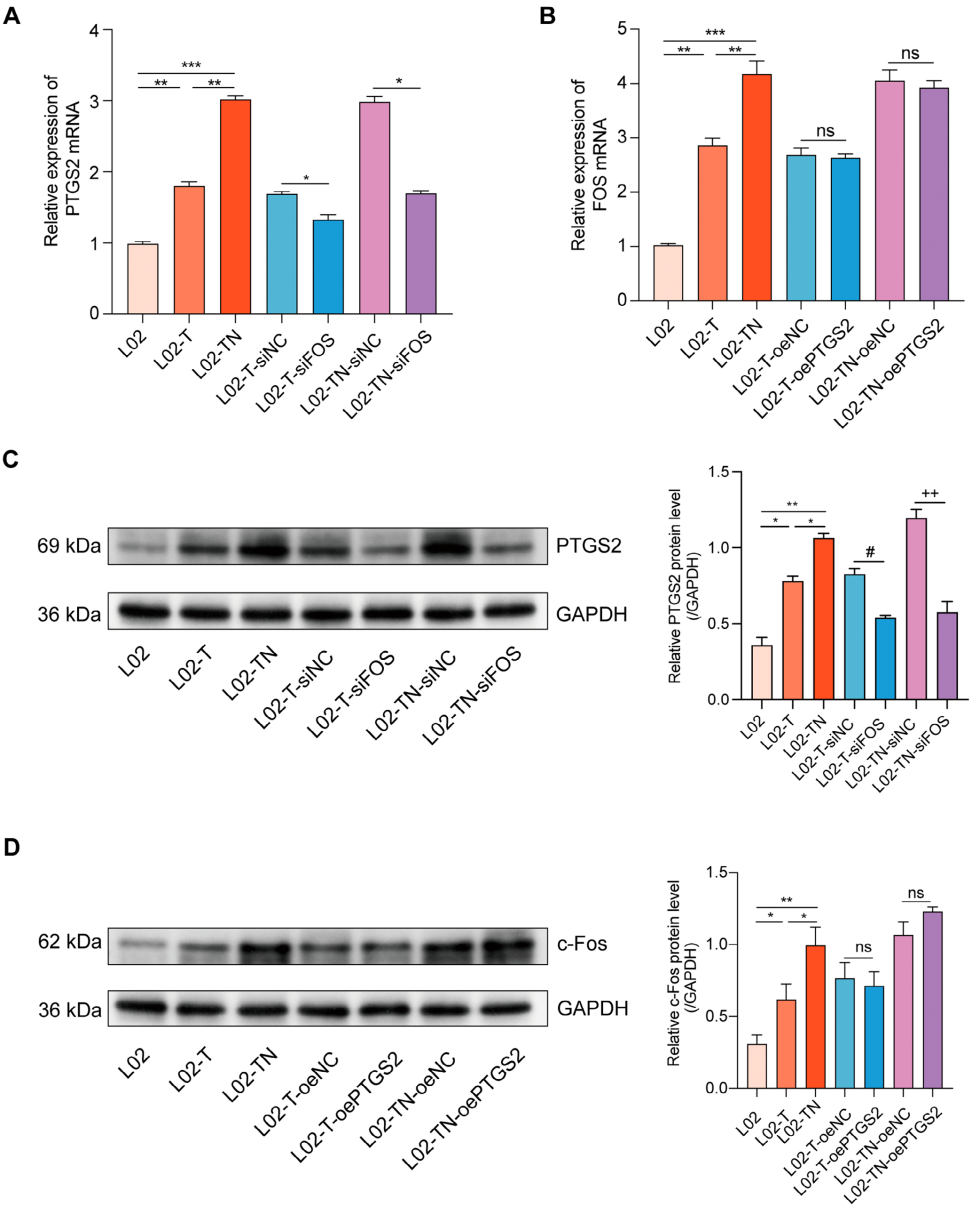
FOS silencing reduces PTGS2 expression, with a stronger effect under T2DM combined with NAFLD. (A) PTGS2 mRNA expression in different treatment groups following FOS silencing. (B) FOS mRNA expression in different groups after PTGS2 overexpression. (C) PTGS2 protein levels in different groups after FOS silencing. (D) PTGS2 protein levels in different groups after PTGS2 overexpression. Data are presented as mean ± standard deviation from three independent experiments. Significance levels: ns, not significant; *p < 0.05; **p < 0.01; ***p < 0.001; ^#^ *p* < 0.05; ^++^ *p* < 0.01.

We further validated the regulatory relationship between FOS and PTGS2 using different treatment conditions in an in vitro model. qPCR results showed that FOS silencing significantly reduced PTGS2 mRNA expression, with a more pronounced inhibitory effect observed in the L02‐TN group compared to the L02‐T group (Figure 8A), suggesting that FOS‐mediated regulation of PTGS2 may be further enhanced during the progression from T2DM to NAFLD. In contrast, PTGS2 overexpression had no effect on FOS expression, supporting the role of FOS as an upstream regulator (Figure 8B). Consistently, Western blot analysis demonstrated that FOS silencing reduced PTGS2 protein levels, whereas PTGS2 overexpression did not affect c‐Fos protein expression (Figure 8C,D).

### 3.9 The FOS‐PTGS2 Axis Promotes Inflammatory and Apoptotic Responses During the Progression From T2DM to T2DM Combined With NAFLD

**FIGURE 9:**
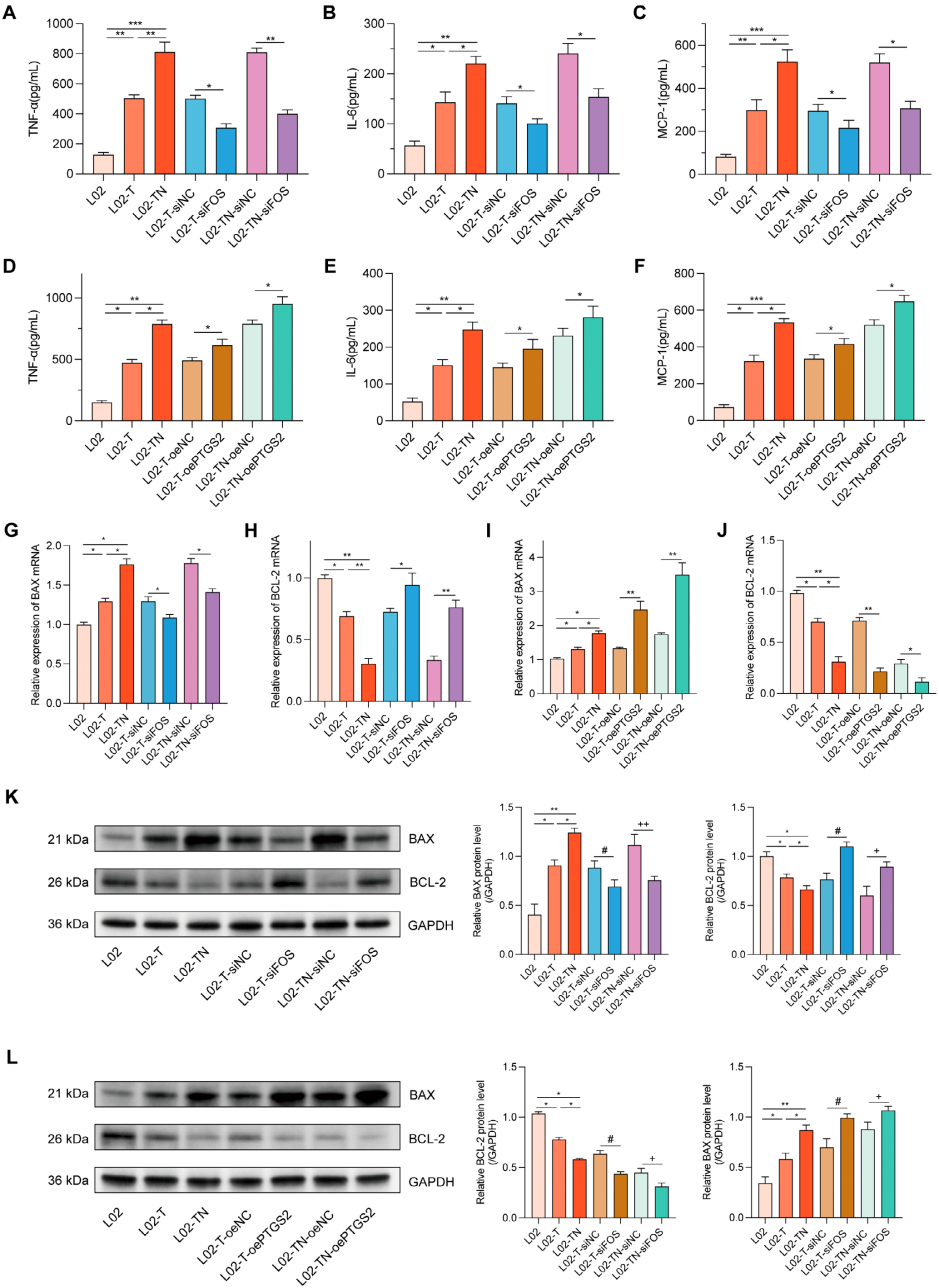
FOS–PTGS2 axis promotes inflammation and apoptosis in T2DM combined with NAFLD progression. (A–C) Protein levels of TNF‐α, IL‐6 and MCP‐1 in different treatment groups following FOS silencing. (D–F) Expression of the same cytokines in different groups after PTGS2 overexpression. (G, H) Effects of FOS silencing on BAX and BCL‐2 mRNA expression. (I, J) Effects of PTGS2 overexpression on BAX and BCL‐2 mRNA expression. (K) Effects of FOS silencing on BAX and BCL‐2 protein levels. (L) Effects of PTGS2 overexpression on BAX and BCL‐2 protein levels. Data are presented as mean ± standard deviation from three independent experiments. Significance levels: ns, not significant; *p < 0.05; **p < 0.01; ***p < 0.001; ^#^ *p* < 0.05; ^+^ *p* < 0.05; ^++^ *p* < 0.01.

To further investigate the functional role of the FOS‐PTGS2 signalling axis in the progression from T2DM to T2DM combined with NAFLD, inflammatory cytokines and apoptosis‐related markers were examined across treatment groups. ELISA results showed that TNF‐α, IL‐6 and MCP‐1 levels were significantly increased in both the L02‐T and L02‐TN groups. Silencing of FOS (L02‐T‐siFOS, L02‐TN‐siFOS) significantly reduced the expression of these cytokines (Figure 9A–C), whereas PTGS2 overexpression (L02‐T‐oePTGS2, L02‐TN‐oePTGS2) further enhanced the inflammatory response (Figure 9D–F). qPCR analysis demonstrated that FOS silencing suppressed the expression of the pro‐apoptotic gene BAX and upregulated the anti‐apoptotic gene BCL‐2 (Figure 9G,H), while PTGS2 overexpression led to the opposite pattern, with increased BAX and decreased BCL‐2 expression (Figure 9I,J). Western blot analysis further confirmed these changes at the protein level: FOS silencing markedly reduced BAX protein levels and increased BCL‐2 levels (Figure 9K), whereas PTGS2 overexpression produced the opposite effects (Figure 9L). Together, these findings indicate that FOS promotes inflammatory cytokine production and apoptosis by upregulating PTGS2, suggesting its critical role in driving pathological progression from T2DM to T2DM combined with NAFLD.

## 4 Discussion

T2DM combined with NAFLD has emerged as a pressing global clinical challenge. T2DM associated NAFLD is now recognised as the fourth leading cause of mortality among chronic liver diseases [32], with a steadily increasing prevalence and more severe metabolic disturbances and multi‐organ complications. However, the underlying pathogenesis remains complex and incompletely understood. Chronic, persistent inflammation has been shown to play a central role in the progression from T2DM to T2DM combined with NAFLD [33, 34]. T2DM is characterised by insulin resistance and pancreatic β‐cell dysfunction [35], both of which are perpetuated by chronic low‐grade inflammation. In T2DM patients, adipose tissue dysfunction leads to excessive release of pro‐inflammatory cytokines and FFAs [36], which circulate to the liver and activate Kupffer cells and infiltrating monocytes [37]. These immune cells further amplify the local inflammatory cascade, forming a vicious cycle of peripheral insulin resistance, hepatic inflammation and metabolic imbalance, thereby accelerating the transition from T2DM to NAFLD [3]. In recent years, increasing attention has been given to the molecular crosstalk between metabolic diseases, highlighting the importance of dissecting shared pathogenic networks to enable early identification and intervention. In this study, we aimed to identify key biomarkers involved in the progression of T2DM to NAFLD and to elucidate their potential molecular mechanisms, providing a theoretical and experimental foundation for early diagnosis and targeted therapy.

Our study identified and validated the regulatory role of the FOS–PTGS2 axis in the progression of T2DM‐associated NAFLD, suggesting that this pathway may exacerbate metabolic dysfunction by promoting inflammatory responses and apoptosis. By integrating peripheral blood RNA‐seq data, WGCNA‐based co‐expression network construction, GO/KEGG enrichment analysis and machine learning algorithms including LASSO and random forest, FOS and PTGS2 were robustly identified as key candidate genes. Previous studies have highlighted the critical role of chronic inflammation in the pathogenesis of both T2DM and NAFLD [38], with sustained elevations in TNF‐α and IL‐6 contributing to insulin resistance and hepatocellular injury [39, 40], while persistent activation of the NF‐κB signalling pathway maintains the inflammatory state [41, 42]. Our enrichment analysis revealed that differentially expressed genes were significantly associated with inflammatory signalling pathways such as TNF, IL‐17 and NF‐κB, as well as biological processes related to apoptosis and oxidative stress, further supporting the involvement of immune inflammation and programmed cell death in the progression from T2DM to NAFLD.

At the single‐cell level, FOS and PTGS2 exhibited distinct expression patterns across immune cell subsets. In peripheral blood, they were predominantly enriched in monocytes, CD8^+^ T cells and Tregs, while in the liver, expression was concentrated in Kupffer cells and endothelial cells—findings consistent with prior studies demonstrating COX‐2 activation in hepatic immune cells [43]. Further transcription factor analysis revealed that FOS, one of the differentially expressed core genes, directly regulates PTGS2 as a downstream target, suggesting a hierarchical transcriptional relationship. Dual‐luciferase reporter assays confirmed that FOS significantly enhances PTGS2 promoter activity, providing additional evidence of this regulatory interaction. This multi‐layered chain of evidence—from differential expression and bioinformatic prediction to functional validation—supports the mechanistic relevance of the FOS‐PTGS2 axis in T2DM‐associated NAFLD. Our findings indicate that this axis may not only serve as a biomarker of disease progression but also mediate key inflammatory and immunopathological changes during disease development.

FOS, as a stress‐inducible transcription factor, has been shown to regulate PTSG2 expression in various pathological contexts [44, 45, 46]. Our results further demonstrate that FOS transcriptionally activates PTGS2, thereby modulating inflammation and apoptosis. The protein product COX‐2, encoded by PTGS2, not only acts as a pro‐inflammatory mediator but also activates downstream signalling pathways including p53, Akt and TNF‐α, promoting ROS generation and resulting in hepatocellular and endothelial cell damage [47, 48]. This mechanism aligns with our in vitro L02 cell model, where high‐glucose and FFA treatment induced FOS–PTGS2‐mediated increases in inflammatory cytokine release and apoptosis. Other studies have reported that AREG enhances iNOS and PTGS2 expression through NF‐κB and MAPK signalling, contributing to hepatic inflammation [49]. Moreover, aberrant PTGS2 expression has been linked to lipotoxicity and insulin secretion defects in pancreatic β‐cells [50] and to lipid accumulation via autophagy inhibition and Wnt signalling activation in adipose tissue [51], highlighting the context‐dependent regulatory roles of the FOS–PTGS2 axis. We also demonstrated a unidirectional regulatory relationship between FOS and PTGS2: silencing FOS significantly downregulated PTGS2 at both mRNA and protein levels, while PTGS2 overexpression had no impact on FOS expression. Dual‐luciferase assays further confirmed FOS as the upstream regulator in this transcriptional cascade. Several studies have shown that PTGS2 contributes to hepatocyte apoptosis by promoting TNF‐α and IL‐6 expression under high‐fat diet conditions [52]. Conversely, in mesenchymal stem cells derived from T2DM patients, impaired COX‐2 expression is associated with reduced immunomodulatory capacity [53]. In our study, the FOS–PTGS2 axis exhibited enhanced pro‐inflammatory and pro‐apoptotic effects under simulated T2DM combined with NAFLD conditions, with the L02‐TN group (treated with both glucose and FFA) showing the most pronounced changes. FOS knockdown significantly reduced PTGS2 expression and downstream inflammatory/apoptotic markers, while PTGS2 overexpression exacerbated these pathological responses, suggesting a synergistic amplification of the axis under lipotoxic stress.

Multiple animal and clinical studies have further confirmed the anti‐inflammatory and organ‐protective effects of COX‐2 inhibitors in the context of T2DM and NAFLD. Selective COX‐2 inhibitors such as celecoxib and nimesulide have been shown to reduce hepatic inflammation and lipid deposition in high‐fat diet‐induced mouse models, while also improving liver–pancreas metabolic function [54, 55]. Clinically, COX‐2 inhibition combined with metformin has been associated with reduced inflammatory markers and hospitalisation rates in patients with T2DM and liver disease [56]. Additional evidence suggests that COX‐2 inhibition may improve glycemic control and reduce the risk of metabolic deterioration [57]. Notably, valdecoxib has been reported to alleviate hepatic steatosis by activating the AMPK/SIRT6 pathway and reducing ER stress through autophagy modulation, providing further insight into potential therapeutic mechanisms [58]. These findings complement our observations that PTGS2 overexpression aggravates inflammation and apoptosis at the cellular level, underscoring the therapeutic potential of targeting the FOS–PTGS2 axis in T2DM‐associated NAFLD.

Despite our findings elucidating the regulatory mechanism and potential therapeutic relevance of the FOS–PTGS2 axis in T2DM‐associated NAFLD, several limitations should be acknowledged. First, the clinical predictive value of FOS and PTGS2 requires further validation in larger, multicentre cohorts. Second, the lack of in vivo studies limits the mechanistic understanding of this axis; future studies involving liver‐specific knockout models are warranted. Finally, although we have preliminarily demonstrated the involvement of this pathway in inflammation and apoptosis, it remains to be clarified whether these effects are mediated via specific immune cell functions or broader metabolic signalling pathways. In summary, our study elucidates the transcriptional regulation of PTGS2 by FOS and its role in the progression of T2DM‐associated NAFLD, providing novel molecular insights that may inform early diagnosis and targeted intervention strategies.

## 5 Conclusion

To conclude, this study demonstrates that FOS promotes the progression from T2DM to T2DM combined with NAFLD by transcriptionally activating PTGS2, thereby mediating hepatocellular inflammation and apoptosis. These findings provide novel mechanistic insights and potential therapeutic targets for a deeper understanding of the pathology underlying this comorbid condition.

## Author Contributions

**Rong Lin:** data curation (equal), funding acquisition (lead), methodology (lead), writing – original draft (lead). **Leqin Xu:** data curation (equal), writing – review and editing (equal). **Yi Zhou:** software (equal), visualization (equal), writing – review and editing (equal). **Yanjing Fan:** formal analysis (equal), writing – review and editing (equal). **Huan Xie:** formal analysis (equal), software (equal), writing – review and editing (equal). **Wanzhang Li:** investigation (equal), resources (equal), writing – review and editing (equal). **Tianchi Hu:** conceptualization (equal), supervision (equal), writing – review and editing (equal). **Chao Liu:** conceptualization (equal), supervision (equal), writing – review and editing (equal).

## Funding

This work was supported by grants from the Natural Science Foundation of Xiamen, China (Grant 3502Z20227356).

## Ethics Statement

All participants were fully informed of the study procedures, potential risks and benefits prior to enrollment and provided written informed consent. The study protocol was reviewed and approved by the Ethics Committee of Xiamen Hospital of Traditional Chinese Medicine (Approval No. 2024‐k004‐01).

## Conflicts of Interest

The authors declare no conflicts of interest.

## Supporting information


**Figure S1:** Identification and annotation of PBMC cell populations in T2DM by single‐cell RNA sequencing. (A) Violin plots showing the distributions of quality‐control metrics, including nFeature_RNA, nCount_RNA, percent.mt and percent.HB, across the annotated PBMC cell populations. (B) PCA and Harmony embeddings of PBMC cells coloured by group (Normal and T2DM) and sample, showing the cellular distribution before and after batch‐effect correction/integration. (C) Clustree plot showing the relationships and stability of cell clusters across different clustering resolutions, which was used to determine the optimal resolution for downstream analysis. (D) Dot plot of canonical marker genes used for cell‐type annotation.
**Figure S2:** Single‐cell transcriptomic landscape and cell‐type annotation of liver tissue from patients with NAFLD. (A) Violin plots summarising the distributions of single‐cell quality‐control metrics, including nFeature_RNA, nCount_RNA, percent.mt and percent.HB, across the annotated hepatic cell populations. (B) Two‐dimensional visualisation of liver‐derived cells by PCA and Harmony integration, coloured according to group (Normal and NAFLD) and sample origin, illustrating the overall cellular structure before and after data integration. (C) Clustering tree analysis across a range of resolution parameters, showing the hierarchical relationships and transition patterns of cell clusters and supporting the selection of an appropriate clustering resolution for subsequent analyses. (D) Dot plot displaying the expression patterns of representative marker genes across major liver cell populations.

## Data Availability

The datasets used in this study are available from the corresponding author upon reasonable request. Additionally, the data supporting the findings of this study are accessible on the GEO database (http://www.ncbi.nlm.nih.gov/geo).
