# Supplementary material for: Integrated Bulk and Single‐Cell RNA‐Seq Analysis Reveals Transcriptional Activation of PTGS2 by FOS in Progression From T2DM to T2DM‐Associated NAFLD
Source: J Cell Mol Med. 2026 May 10;30(9):e71182. doi: 10.1111/jcmm.71182 (PMC13158135; doi:10.1111/jcmm.71182)
Supplement: Supplementary file 1 — Figure S1: Identification and annotation of PBMC cell populations in T2DM by single‐cell RNA sequencing. (A) Violin plots showing the distributions of quality‐control metrics, including nFeature_RNA, nCount_RNA, percent.mt and percent.HB, across the annotated PBMC cell populations. (B) PCA and Harmony embeddings of PBMC cells coloured by group (Normal and T2DM) and sample, showing the cellular distribution before and after batch‐effect correction/integration. (C) Clustree plot showing the relationships and stability of cell clusters across different clustering resolutions, which was used to determine the optimal resolution for downstream analysis. (D) Dot plot of canonical marker genes used for cell‐type annotation. Figure S2: Single‐cell transcriptomic landscape and cell‐type annotation of liver tissue from patients with NAFLD. (A) Violin plots summarising the distributions of single‐cell quality‐control metrics, including nFeature_RNA, nCount_RNA, percent.mt and percent.HB, across the annotated hepatic cell populations. (B) Two‐dimensional visualisation of liver‐derived cells by PCA and Harmony integration, coloured according to group (Normal and NAFLD) and sample origin, illustrating the overall cellular structure before and after data integration. (C) Clustering tree analysis across a range of resolution parameters, showing the hierarchical relationships and transition patterns of cell clusters and supporting the selection of an appropriate clustering resolution for subsequent analyses. (D) Dot plot displaying the expression patterns of representative marker genes across major liver cell populations. [file JCMM-30-e71182-s001.docx]

**Integrated bulk and single-cell RNA-seq analysis reveals transcriptional activation of PTGS2 by FOS in progression from T2DM to T2DM-associated NAFLD**

Rong Lin^1,3^, Leqin Xu^2^, Yi Zhou^3^, Yanjing Fan^3^, Huan Xie^3^, Wanzhang Li^3^, Tianchi Hu^3^*, Chao Liu^1^*

^1^Department of Endocrinology, Affiliated Hospital of Integrated Traditional Chinese and Western Medicine for Nanjing University of Chinese Medicine, Nanjing, China.

^2^Xiamen Hospital of Traditional Chinese Medicine (Xiamen Hospital, Beijing University of Chinese Medicine), Xiamen, China.

^3^Department of Endocrinology, Xiamen Hospital of Traditional Chinese Medicine (Xiamen Hospital, Beijing University of Chinese Medicine), Xiamen, China.

*Correspondence: Chao Liu, Email: profliuc@163.com, liuchao@nfmcn.com; Tianchi Hu，Email: hugogob@sina.com.


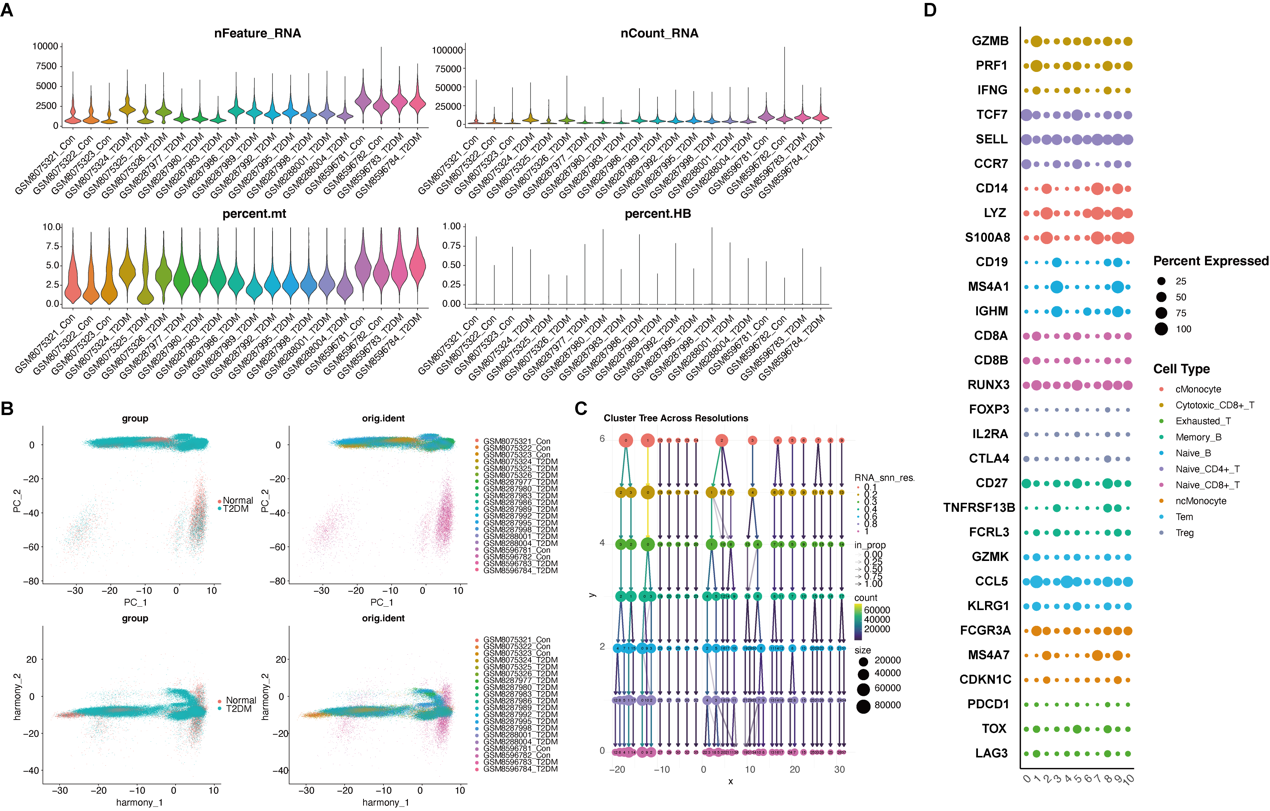


**Supplementary Figure 1** Identification and annotation of PBMC cell populations in T2DM by single-cell RNA sequencing. (A) Violin plots showing the distributions of quality-control metrics, including nFeature_RNA, nCount_RNA, percent.mt, and percent.HB, across the annotated PBMC cell populations. (B) PCA and Harmony embeddings of PBMC cells colored by group (Normal and T2DM) and sample, showing the cellular distribution before and after batch-effect correction/integration. (C) Clustree plot showing the relationships and stability of cell clusters across different clustering resolutions, which was used to determine the optimal resolution for downstream analysis. (D) Dot plot of canonical marker genes used for cell-type annotation.


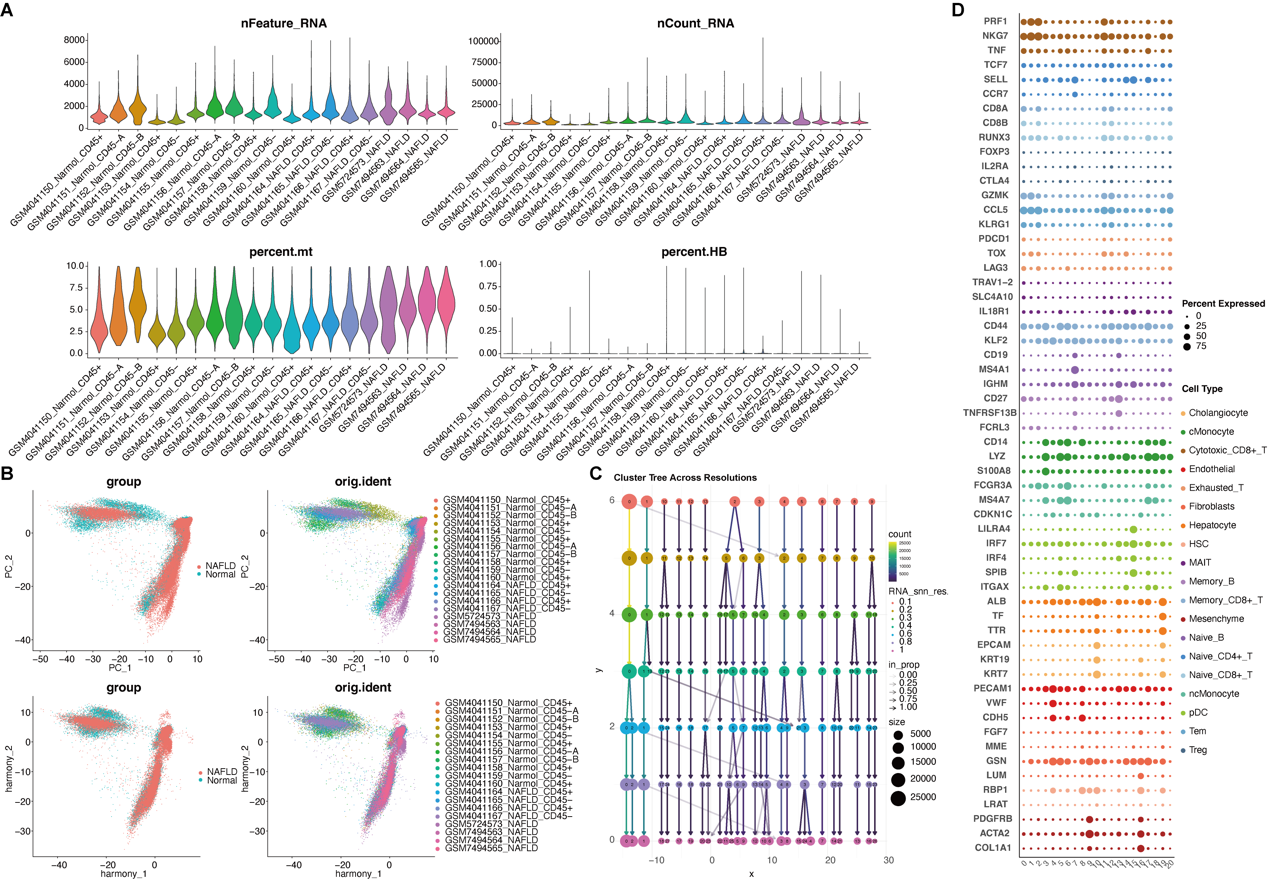


**Supplementary Figure 2** Single-cell transcriptomic landscape and cell-type annotation of liver tissue from patients with NAFLD. (A) Violin plots summarizing the distributions of single-cell quality-control metrics, including nFeature_RNA, nCount_RNA, percent.mt, and percent.HB, across the annotated hepatic cell populations. (B) Two-dimensional visualization of liver-derived cells by PCA and Harmony integration, colored according to group (Normal and NAFLD) and sample origin, illustrating the overall cellular structure before and after data integration. (C) Clustering tree analysis across a range of resolution parameters, showing the hierarchical relationships and transition patterns of cell clusters and supporting the selection of an appropriate clustering resolution for subsequent analyses. (D) Dot plot displaying the expression patterns of representative marker genes across major liver cell populations.
